# Research Advances on Swine Acute Diarrhea Syndrome Coronavirus

**DOI:** 10.3390/ani14030448

**Published:** 2024-01-30

**Authors:** Chuancheng Liu, Weili Huang, Xinyan He, Zhihua Feng, Qi Chen

**Affiliations:** 1 College of Life Science, Fujian Normal University, Fuzhou 350117, China; qsx20211374@student.fjnu.edu.cn (C.L.); qsx20211392@student.fjnu.edu.cn (W.H.); qsx20221410@student.fjnu.edu.cn (X.H.); 2 Fujian Key Laboratory of Innate Immune Biology, Biomedical Research Center of South China, Fujian Normal University, Fuzhou 350117, China

**Keywords:** swine acute diarrhea syndrome coronavirus, SADS-CoV, zoonosis, virus–host interaction, cross-species transmission

## Abstract

**Simple Summary:**

Swine acute diarrhea syndrome coronavirus is an emerging enteropathogenic coronavirus with high lethality in lactating piglets and strong cross-species transmission ability. As it is a new threat to the swine industry, research and understanding of this virus are still in the early stages. There are currently no commercially available vaccines that prevent swine acute diarrhea syndrome coronavirus (SADS-CoV). In this review, we systematically analyzed the structural components of the virus, input and possible modes of transmission, and the cross-species transmissibility of the virus to characterize its risk level. We also summarized host-dependent factors in the virus infection process, as well as the viral regulation of host cell life processes. Finally, we discussed preventive and therapeutic measures that can be adopted in the future. Through this review, we aim to contribute to a better understanding of this porcine enterovirus and to subsequent research.

**Abstract:**

Swine acute diarrhea syndrome coronavirus (SADS-CoV) is a virulent pathogen that causes acute diarrhea in piglets. The virus was first discovered in Guangdong Province, China, in 2017 and has since emerged in Jiangxi, Fujian, and Guangxi Provinces. The outbreak exhibited a localized and sporadic pattern, with no discernable temporal continuity. The virus can infect human progenitor cells and demonstrates considerable potential for cross-species transmission, representing a potential risk for zoonotic transmission. Therefore, continuous surveillance of and comprehensive research on SADS-CoV are imperative. This review provides an overview of the temporal and evolutionary features of SADS-CoV outbreaks, focusing on the structural characteristics of the virus, which serve as the basis for discussing its potential for interspecies transmission. Additionally, the review summarizes virus–host interactions, including the effects on host cells, as well as apoptotic and autophagic behaviors, and discusses prevention and treatment modalities for this viral infection.

## 1. Introduction

Coronaviruses (CoVs) are the largest positive-sense RNA viruses, belonging to the family *Coronaviridae* and the order *Nidovirales*. The virus was named after its specialized crown-like structure, as observed in electron microscope images [1,2]. Coronaviruses have the largest genome of any known RNA virus, with a genome size of about 26–32 KB [3]. All coronaviruses have a similar organization and expression of their genomes [2]. First, there is the open reading frame (ORF) 1a/b, which can encode 16 non-structural proteins and occupies about two-thirds of the genome length [4], starting from the 5′ end, followed by the structural proteins spike (S), envelope (E), membrane (M), and nucleocapsid (N), and finally the ORF, expressing non-structural proteins near the 3′ end [5]. Based on the serology and the genome, the subfamily of coronaviruses can be divided into four genera: α, β, γ, and δ [6]. The coronavirus genome encodes various proteins, including structural, non-structural, and accessory proteins. Coronaviruses, like other RNA viruses, have a high mutation rate and a strong tendency to reorganize despite possessing a proofreading-active ribonucleic acid exonuclease (ExoN)-containing non-structural protein (nsp14) [7]. These characteristics allow them to overcome host species barriers and to adapt to new hosts. This has resulted in repeated demonstrations of their zoonotic and terrestrial transmission capabilities, such as those of COVID-19, Severe Acute Respiratory Syndrome Coronavirus (SARS-CoV), and Middle East Respiratory Syndrome Coronavirus (MERS-CoV), which originated in bats and were transmitted to humans via intermediate mammalian hosts [8]. Because virus epidemics pose potential risks to human health and economic and social development, it is crucial to recognize the virus and implement proactive measures to reduce or eliminate its impact.

Porcine coronavirus infections have resulted in significant economic losses in the swine farming sector [9]. For example, in October 2010, coronavirus infections in pigs cumulatively led to the deaths of more than one million piglets in China [10]; the 2013 outbreak of PEDV in the United States, Canada, and Mexico killed more than eight million piglets in the United States alone [11]; and in the first half of 2017, the first outbreak of SADS-CoV led to the deaths of more than 20,000 piglets [12]. These infections are caused by six different coronaviruses: porcine epidemic diarrhea virus (PEDV), porcine transmissible gastroenteritis virus (TGEV), porcine delta coronavirus (PDCoV), porcine acute diarrheal syndrome coronavirus (SADS-CoV), porcine hemagglutinating encephalomyelitis virus (PHEV), and porcine respiratory coronavirus (PRCV) [13]. These viruses belong to three genera within the subfamily Orthocoronaviridae. PEDV, TGEV, SADS-CoV, and PRCV belong to the genus α-coronavirus, whereas PHEV and PDCoV belong to the genera β-coronavirus and δ-coronavirus, respectively. Different genera of viruses typically induce distinct clinical manifestations. Pigs infected with α-coronaviruses typically exhibit enteritis and watery diarrhea. These viruses are commonly referred to as porcine enteric coronaviruses (PEC) [14,15]. In contrast, PHEV causes encephalomyelitis, whereas PRCV causes respiratory disease [16,17]. Among the six porcine coronaviruses mentioned, PHEV and PRCV primarily infect individuals with occult infections, with or without mild clinical signs [18,19]. PECs are a significant cause of economic loss and the transmission of epidemics in farming. Since the last century, TGEV and PEDV have caused substantial economic losses in swine farming worldwide. PDCoV and SADS-CoV are emerging PECs that were discovered in 2014 (USA) and 2017 (China), respectively [20].

SADS-CoV, a novel porcine α-coronavirus, was first discovered in Guangdong Province, China, in 2017 [21]. SADS-CoV is closely related to bat coronavirus HKU2 [22], which was isolated by the University of Hong Kong in 2006. It originates from *Rhinolophus sinicus* and has a genome length of approximately 27.2 kilobases [23]. As of 2023, SADS-CoV has caused outbreaks in Guangdong and its neighboring provinces, including Fujian, Guangxi, and Jiangxi. Infected pigs exhibit clinical signs such as acute diarrhea and vomiting, along with high mortality rates in piglets. Although the scale of outbreaks caused by this virus has not yet reached pandemic levels, it is still endemic to specific regions. Studies have consistently shown that SADS-CoV, with its unique structure, is at risk of evolving into a zoonotic virus [24,25,26]. The study of SADS-CoV is critical for the surveillance and control of animal diseases, while also enhancing our understanding of coronaviruses by identifying their unique characteristics. This review will provide a summary and discussion of the research progress on SADS-CoV, focusing on its evolutionary features, cross-species transmissibility, zoonotic potential, key host factors, and prevention and treatment methods.

## 2. The Outbreak and Evolution of Swine Acute Diarrhea Syndrome Coronavirus (SADS-CoV)

**Figure 1:**
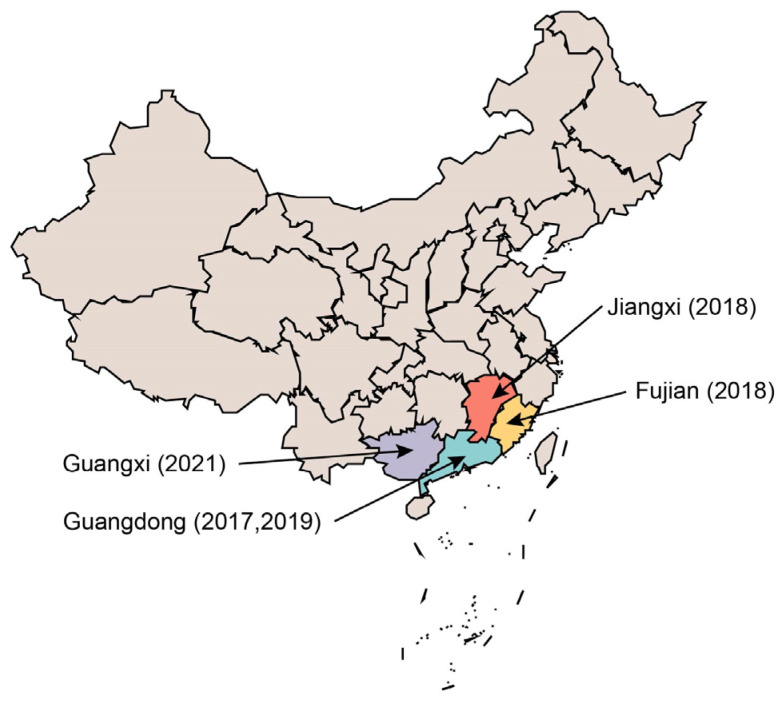
Distribution of SADS-CoV-induced outbreaks by time and location. Note: SADS-CoV was first discovered in Guangdong Province, China. Since its discovery, it has caused epidemics in Guangdong, Jiangxi, Fujian, and Guangxi.

In February 2017, an outbreak of severe diarrhea in PEDV-vaccinated pigs occurred in Guangdong Province, China [27]. All affected pigs showed clinical signs of severe watery diarrhea, and the outbreak was caused by a novel α-coronavirus belonging to the same branch as HKU2, which was named porcine enteric α-coronavirus (PEAV) [28]. In 2018, the SADS-COV strain was detected in fecal samples of diarrheic piglets from seven farms in Fujian Province and was named CH/FJWT/2018 (GenBank No. MH615810) [29]. In February 2019, an outbreak occurred in Guangdong Province that resulted in swine diarrheal deaths, and a virological investigation identified the SADS-CoV virus as the cause, which was named CN/GDLX/2019 (GenBank No. MK651076). In 2021, an outbreak of severe acute diarrhea, vomiting, and weight loss in piglets caused by the SADS-CoV virus was reported in Guangxi Province, with a mortality rate of up to 100% [30] (Figure 1). The strain responsible for the outbreak was named SADS-CoV/Guangxi/2021 (GenBank No. ON911569) [30]. Currently, the virus has only been reported in Guangdong, Fujian, Guangxi, and Jiangxi provinces in China, with no cases reported in other provinces or elsewhere globally [31]. The virus outbreaks are primarily concentrated in the southern region of China, with a localized distribution and limited spread. The virus may be more susceptible to the high temperatures and humidity in the southern region, limiting its spread.

**Figure 2:**
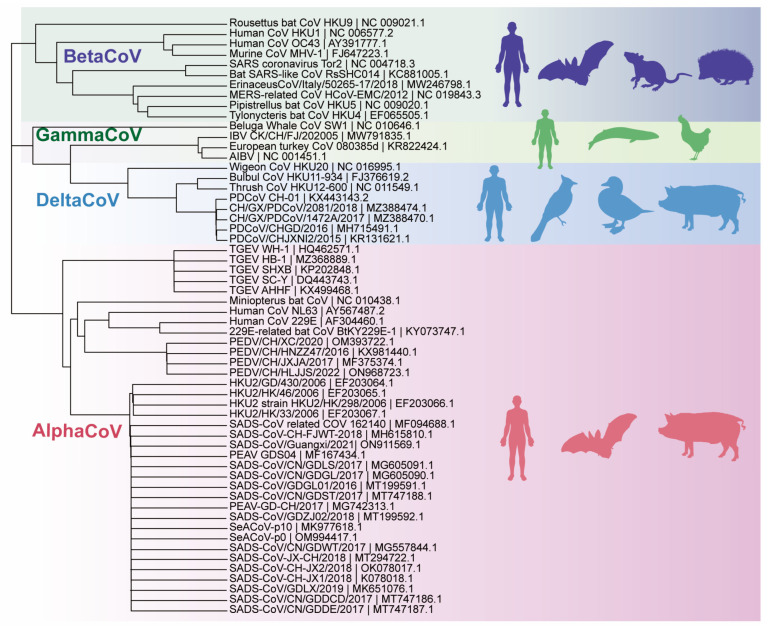
Phylogenetic tree analysis based on the genomic sequence of SADS-CoV. Note: The data in the phylogenetic tree come from the full-length sequence of the virus in national center for biotechnology information (NCBI) and the tree was drawn using MEGAX version 10.2.6. The phylogenetic tree table shows that SADS-CoV belongs to the α-coronavirus and is closely related to HKU2; the SADS-CoV strains detected in different regions from 2017 to 2021 are all in the same branch.

All identified sequences of this virus belong to the sublineage “SeACoV/PEAV/SADS-CoV” and have a sequence identity of >98.4%. The reported strains of SADS-CoV are genetically similar to the bat “HKU2-like-CoV” sublineage. The SADS-CoV shares a 96–98% sequence identity with the SADS-related coronavirus (SADSr-CoV) detected in anal swabs collected from different *Rhinolophus* species in Guangdong Province from 2013 to 2016,which further suggests that SADS-CoV may have originated in bats [12,27,32]. SADS-CoV and HKU2 are classified as α-coronaviruses based on their complete genomes (Figure 2), but phylogenetic analyses of their spike proteins have revealed that they belong to the β-coronavirus group [12,27,33,34,35,36]. SADS-CoV is believed to have emerged through a recombination between the α-coronavirus and an unrecognized β-coronavirus S gene [22,32,33].

## 3. Structure of the Swine Acute Diarrhea Syndrome Coronavirus (SADS-CoV) Genome and Its Functions

**Figure 3:**
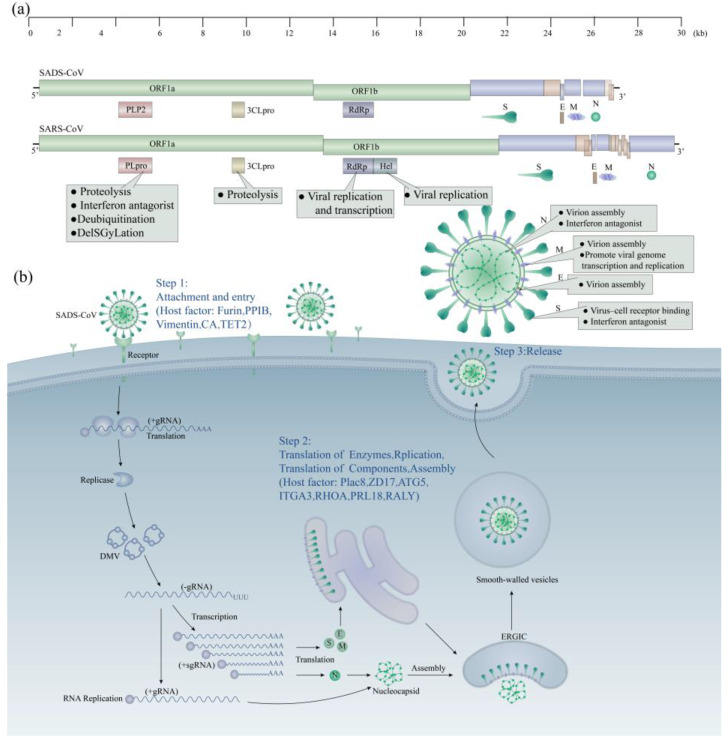
(**a**) Genome and viral structure of SADS-CoV; (**b**) cellular invasion process of SADS-CoV and host key factors. Note: (**a**) the total length of the SADS-CoV genome is approximately 27.2 kb. The viral genome encodes four structural proteins, as well as multiple nonstructural proteins. The proteins mentioned above involve virus invasion, assembly, immune evasion, replication, transcription, and other processes. (**b**) The currently identified critical factors of the SADS-CoV host are closely related to the infection and proliferation of the virus.

The SADS-CoV genome is approximately 27.2 kb in length and comprises the following components arranged sequentially: 5′-untranslated region (UTR), open reading frame (ORF) 1a/1b, spike protein (S), ORF3, envelope protein (E), membrane protein (M), nuclear coat protein (N), NS7a/7b (putative accessory genes), and 3-′UTR. ORF1a and ORF1b, which encode non-structural proteins in the SADS-CoV genome, constitute approximately two-thirds of the genome, similar to other coronaviruses [33,37,38]. After infecting the host cells, the virus uses its genomic RNA as messenger RNA to translate ORF1a and ORF1b through host ribosomes, resulting in the production of polyprotein 1a (pp1a) and polyprotein 1b (pp1b) [39,40]. These polyproteins are subsequently cleaved by two virus-encoded protein hydrolases, PLP2 and 3CLPro, to yield 16 non-structural proteins. SADS-CoV PLP2 is functionally and operationally similar to SARS-CoV PLPro [41]. SADS-CoV PLP2 exhibits cleavage activity on nsp1 and peptides mimicking nsp2|nsp3 cleavage sites, and functions as a regulatory protein in the formation of the SADS-CoV replicase complex (RC). PLP2 exhibits deubiquitinating and deISGynating [42,43] activities in vitro, and functions as an interferon inhibitory molecule that suppresses the host’s natural immune response [44]. Enzymatically cleaved non-structural proteins can co-localize with the Golgi, endoplasmic reticulum, nucleus, and microtubule proteins, indicating their functional diversity in various cellular processes. For example, nsp9 co-localizes with microtubule proteins, suggesting its potential involvement in cellular morphological alterations following SADS-CoV infection [45] (Figure 3).

The coronavirus spike glycoprotein (S) comprises the S1 and S2 subunits [46]. During viral infection, the S1 subunit binds to a receptor on the cell membrane, followed by the fusion of the S2 subunit with the cell membrane, facilitating viral entry [47,48,49]. Therefore, the S subunit plays a key role in determining the host range and tissue tropism of coronaviruses [50,51]. SADS-CoV S (1130 amino acid residues) and HKU2 S (1128 amino acid residues) are two of the shortest coronavirus spike glycoproteins [52]; they share a high amino acid identity of 86% and exhibit structural similarities. However, their amino acid identity with other known coronavirus spike glycoproteins is less than 28% [12,34,36], suggesting that the S of HKU2 and SADS-CoV possess unique structural properties [53]. SADS-CoV, an α-coronavirus, shares structural similarities with β-coronaviruses in its S1 subunit. The S1 subunit can be divided into two distinct domains, the N-terminal domain (NTD) and C-terminal domain (CTD) [54], for the recognition of cell surface carbohydrates and specific binding to cellular protein receptors, respectively. The receptor-binding domain (RBD) of most coronavirus S proteins, including SARS-CoV and MERS-CoV, is located in the CTD. A small percentage of β-coronaviruses possess RBDs that are located within the NTD, e.g., murine hepatitis virus [55]. The NTD and CTD of α-coronaviruses can be further divided based on their different configurations. α-coronaviruses can be categorized into two groups based on the distribution of their disulfide bonds, which can be differentiated by the structural variations observed in galactose glucagon-like β-sandwich folds within their NTD structures [53]. The structural similarities between the NTDs of HKU2 and SADS-CoV, and those of HCoV-NL63 and PEDV, which are classified as subtype I, suggest a possible evolutionary relationship between these coronaviruses. The NTDs of β-coronaviruses, including MERS-CoV [56,57], SARS-CoV [58], and MHV [59,60], share similarities with subtype I in terms of topology, the distribution of disulfide bonds, and structural features, which may influence their immune evasion and receptor-binding abilities [61,62,63]. The α-coronavirus CTD structure can be categorized into two groups, single-layer and double-layer β-folded cores, based on the number of twisted β-folded cores. Most α-coronaviruses, such as PEDV and TGEV, have a two-layered β-folding structure. However, the CTD cores of SADS-CoV and HKU2 have a single twisted β-folded core and exhibit a structural similarity to the conserved CTD core of β-coronaviruses [64,65]. In summary, the idea that SADS-CoV originated from the recombination of α-coronavirus with an unidentified β-coronavirus S gene [33,34,66] was further supported by the unique structure of the SADS-CoV S protein.

The unique structure of the SADS-CoV S protein enhances its ability to evade the immune system [67]. The S monomer forms a compact stack of spike protein trimers, reducing the surface area exposed to the host immune system and the immunogenicity. Additionally, the SADS-CoV NTD and CTD structural domains maintain a closed conformation when they are not bound to the host receptor [68]. They adopt an open conformation only after binding to the host receptor. This transition from a closed to an open conformation is thought to minimize the exposure of the RBD structure to the immune system. Furthermore, the surface of the spike protein trimer of SADS-CoV has a 45 N-linked glycan distribution [67]. Epitope masking by glycan shielding promotes the immune escape of SADS-CoV by reducing receptor-binding motif (RBM) exposure [53].

The coronavirus membrane protein (M) is highly conserved, non-glycosylated, and membrane-associated and is the most abundant structural protein in coronaviruses that plays a crucial role in viral assembly and release, as well as in the host’s innate immune response [69]. The protein comprises a short N-terminal outer domain, three transmembrane (TM) domains, and a C-terminal inner domain. The SARS-CoV-2 M protein inhibits the production of type I and type III interferons triggered by the cytoplasmic double-stranded RNA (dsRNA)-sensing pathway [70,71]. The SADS-CoV M protein interacts with host proteins in various cellular compartments, including the cytoplasm, ribosomes, and biological membranes. The M protein is believed to use the membrane structure of the host cell to aid in the assembly and release of progeny virus particles, aligning with the established function of the coronavirus M protein [72,73]. In addition, the SADS-CoV M protein affects the host’s ribose biosynthesis and function, multiple metabolic pathways, the PI3K-ATK signaling pathway, and apoptosis [70]. The M protein accumulates in the nucleus and nucleolus at the early stage of viral infection and persists in the nucleolus throughout the course of infection, with the main function being the inhibition of host cell gene transcription and translation [70]. This ensures that the viral genome has sufficient time in the cytoplasm for replication and transcription.

Coronavirus envelope proteins (E) are short polypeptides containing at least one TM structural domain and two to three near-membrane cysteine clusters [74]. These proteins are involved in viral morphogenesis and host tropism, and their absence can result in the formation of abnormal or attenuated viral particles. Additionally, these proteins have been implicated in the induction of apoptosis [75]. The intracellular distribution of the SADS-CoV E protein was observed in late endosomes and lysosomes. Coronavirus envelope proteins are important for increasing the membrane permeability to ions and are involved in viral morphogenesis and assembly [76,77]. These findings have led to the conclusion that the maturation of SADS-CoV occurs in late endosomes [45,78,79].

The nucleocapsid protein (N) is highly abundant in coronaviruses [80]. During viral replication, the N protein binds to viral genomic RNA [81], resulting in the formation of a helical nucleocapsid [80]. Research has shown that during SADS-CoV infection, the N protein localizes to the cytoplasm and nucleolus [82]. Although the cytoplasmic localization of the protein reflects its consistent function and location, further investigations are required to determine its function in the nuclei of infected host cells. Additionally, the N protein enhances the immune escape ability of the virus by directly blocking the interaction between TRAF3 and TBK1, thereby inhibiting IFN-β production [83,84].

The accessory proteins present in coronaviruses are genus-specific and vary in number across coronaviruses. The predicted sequences of these proteins display minimal homology, even within the same genus [23]. The SADS-CoV genome comprises three putative helper genes: ORF3, NS7a, and NS7b [12,31,85]. Two accessory proteins have been studied, NS7a and NS7b, and antibodies targeting NS7a have been prepared [23]. In addition, the NS7a gene undergoes a large substitution during the middle and late stages of SADS-CoV/CN/GDWT/2017 transmission, resulting in a change in its virulence [85]. At present, the role of SADS-CoV NS7a in viral replication is uncertain, but it is speculated to contribute to the pathogenesis of the virus [23]. However, further investigation is required to confirm these findings.

## 4. Swine Acute Diarrhea Syndrome Coronavirus (SADS-CoV) Importation and Cross-Species Transmission

**Figure 4:**
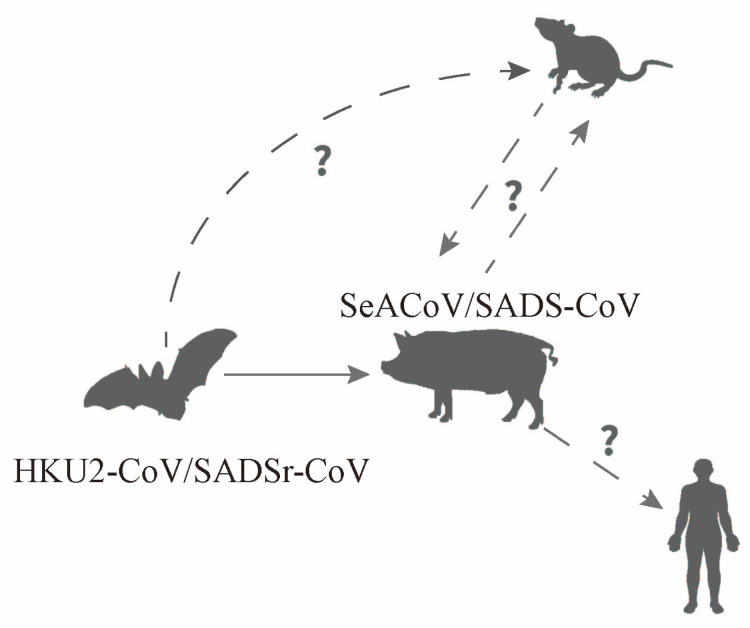
Importation and potential transmission routes of SADS-CoV [12,27,33,34,35,36]. Note: SADS-CoV is currently believed to have evolved from a virus carried by bats. It is speculated that SADS-CoV is transmitted from rodents to pigs, causing an epidemic. Humans may be potential hosts for this virus. The solid lines in the figure represent the currently identified and widely recognized importation or overflow pathways of the SADS-CoV virus; the dotted lines and “?” represent potential transmission pathways that may exist.

The initial spread of SADS-CoV in the first outbreak site is believed to have occurred when HKU2-like-CoV-contaminated bat guano was carried by other animals, such as rodents [53] (Figure 4). A recombination event then occurred in an intermediate host, resulting in the accidental importation of the virus into pig farms [12,27,33,34,35,36]. Once introduced, the virus primarily spreads among swine populations via the fecal–oral route. Co-infection with other porcine epidemic viruses, including PEDV, PDCoV, and TGEV, is often associated with SADS-CoV transmission among swine populations. Dual infection with SADS-CoV and PEDV is the most prevalent, and PEDV-infected piglets in particular are more susceptible to SADS-CoV infection [86,87]. This phenomenon may be attributed to the antibody produced by the individual in response to viral infection, which facilitates the entry of the virus into target cells during SADS-CoV infection through the antibody-dependent enhancement (ADE) effect. This process, which has been observed in numerous viral infections across several families and genera, including coronaviruses, increases the rate of infection [88]. Further confirmation is required to establish the role of ADE in SADS-CoV infection [88,89]. Since viruses with ADE effects tend to exhibit a preference for colonizing macrophages and a tendency to cause persistent infections in the host, further research in this area will contribute to the improved control and treatment of these viruses.

SADS-CoV can enter host cells through three mechanisms, cell membrane caveolae-like depressions, lattice protein-mediated cytophagy, and receptor-independent mediated macroautophagy, indicating a broad cytophilicity [28,90]. Numerous studies have demonstrated that SADS-CoV can replicate in various cells. The in vitro cytophilic range of SADS-CoV is wider than that of other coronavirus cell line susceptibility tests, such as SARS-CoV and MERS-CoV, implying a high risk of cross-species transmission. Furthermore, there is a possibility for the risk of SADS-CoV cross-species transmission, since SADS-CoV infects various mammalian cell lines, including in humans, mice, monkeys, and the chicken fibroblast line DF-1 [25,26,91]. It has been demonstrated that SADS-CoV can infect avian species [91]. The infected chickens displayed mild respiratory clinical signs and the presence of the virus in the lungs and spleens [91]. In addition, there is an increased risk of zoonotic transmission. SADS-CoV has a higher replication efficiency in human cell lines than porcine cell lines, also indicating a potential risk of cross-species transmission to humans. The SADS-CoV virus can infect the human upper respiratory tract (Hep2) and lower respiratory tract (A549) cell lines [25]. The virus also replicates efficiently in various primary human lung cells, including microvascular endothelial cells (MVE), fibroblasts (FB), human nasal epithelial cells (HNE), and human airway epithelial cells (HAE), as well as primary human intestinal cells [26]. This finding supports the high risk of zoonotic transmission of SADS-CoV by effectively eliminating the influence of innate immunity and other antiviral genes in immortalized cells [25,26,92] (Table 1).

## 5. Swine Acute Diarrhea Syndrome Coronavirus (SADS-CoV) and the Occurrence of Apoptosis and Autophagy

**Figure 5:**
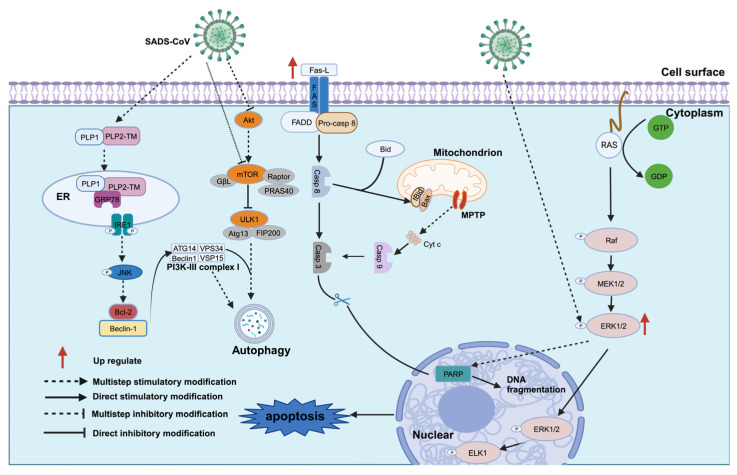
SADS-CoV infection induces apoptosis and complete autophagy. Note: SADS-CoV infection triggers two apoptotic pathways, engaging both the exogenous death receptor and the endogenous mitochondrial pathways. Additionally, SADS-CoV can induce apoptosis in host cells through the RAS-Raf-MEK1/2-ERK1/2 signaling pathway. SADS-CoV induces autophagy via the IRE1–JNK–Beclin1 and AKT/mTOR signaling pathways.

SADS-CoV triggers apoptosis and autophagy to facilitate replication [93,94]. Apoptosis is a programmed cell death process that regulates cell death, and can be categorized into exogenous and endogenous apoptosis, depending on the triggering conditions [95]. Apoptosis also serves as a crucial antiviral mechanism in host cells [96,97], and SADS-CoV-infected cells exhibit significant apoptosis, characterized by cytoplasmic membrane bulging, nuclear fragmentation, and apoptotic vesicle formation. Biochemical analysis revealed the presence of nucleosome-sized DNA ladders, and terminal deoxynucleotidyltransferase-mediated dUTP nick-end Labeling (TUNEL) staining detected DNA double- or single-strand breaks [93]. Mechanistically, SADS-CoV infection activates two apoptotic pathways involving caspase-8 and caspase-9: the exogenous death receptor and the endogenous mitochondrial pathways, respectively. Activation occurs in a time- and dose-dependent manner, and there is evidence of crosstalk between both activation pathways [93]. The inhibition of poly ADP-ribose polymerase (PARP) cleavage in SADS-CoV-infected cells significantly inhibits SADS-CoV infection [93]. The increased expression of the death ligand FasL on infected cell surfaces facilitates the binding of the FasL homotrimeric complex to its receptor Fas, leading to the formation of the death-inducing signaling conduction complex (DISC) [98]. Pro-caspase-8 in DISC undergoes self-cleavage into active caspase-8 [99]. This activated caspase-8 then initiates an exogenous apoptotic cascade by cleaving caspase-3 [100]. The activated caspase-8 cleaves Bid, a member of the Bcl-2 family that regulates apoptosis, resulting in the formation of the smaller molecule, tBid [101]. tBid then activates pro-apoptotic Bax and allows its translocation into the mitochondria of infected cells, leading to an increase in the permeability of the outer mitochondrial membrane (MOMP). The resulting mitochondrial Cytc release triggers apoptosis through the activation of the endogenous mitochondrial pathway-mediated apoptosis via caspase-9 [93]. However, MOMP does not induce the release of the pro-apoptotic AIF [102,103] protein from the mitochondria, indicating that SADS-CoV does not activate apoptosis in host cells by delivering AIF to the nucleus for chromosome dissociation [94]. A study found that SADS-CoV-infected host cells activate endogenous mitochondrial pathways in a cyclophilin D (CypD)-dependent manner, based on the mitochondrial permeability transition pore (MPTP) model [104]. Cyclosporin A (CsA) [105], a chemical inhibitor of CypD [106], effectively inhibits SADS-CoV-induced apoptosis [93] (Figure 5).

SADS-CoV induces host cell apoptosis via the RAS-Raf-MEK1/2-ERK1/2 signaling pathway [107], in which ERK1/2 activation is crucial for SADS-CoV replication and is positively correlated with viral dose. In addition, apoptosis is positively correlated with ERK1/2 activation [108]. Inhibiting ERK1/2 activation using specific inhibitors effectively reduces SADS-CoV infection-induced poly(ADP-ribose) polymerase (PARP) cleavage [109,110]. In SADS-COV-infected host cells, ERK1/2 activation is only affected by the virus that initially enters the cell and reaches a maximum over time [111]. The inhibition of ERK1/2 activation using PD98059 and U0126 [112,113], specific inhibitors of the upstream signaling MEK1/2, and siRNAs, to specifically reduce ERK1/2 expression in target cells, decreased SADS-CoV virus production in target cells [108].

Autophagy plays a crucial role in cellular physiological processes, as it breaks down damaged organelles and macromolecules for utilization [114]. Additionally, it plays a role in the response to viruses by activating autophagy, which separates the subviral components from autophagosomes for lysosomal degradation [115,116]. However, certain coronaviruses can promote viral proliferation by preventing the formation of autolysosome and terminating the occurrence of complete autophagy [116,117]. Unlike the above, complete autophagy can promote SADS-CoV proliferation in cells. Rapamycin, an autophagy inducer, promotes SADS-CoV production, whereas the inhibition of autophagy with 3-methylamine (3-MA) and the blockage of autophagosome–lysosome fusion with bafilomycin A1 (BafA1) hinders viral proliferation [118]. Mechanistically, SADS-CoV induces autophagy via the IRE1–JNK–Beclin1 and AKT/mTOR signaling pathways [119]. The PLP2-TM functional domain of the viral NSP3 protein activates the IRE1–JNK–Beclin1 signaling pathway by interacting with GRP78 to induce autophagy [119]. This, in turn, inhibits the phosphorylation of both Akt and mTOR proteins [120], thereby reducing their inhibitory effect on autophagy [118]. SADS-CoV promotes autophagy in the presence of viral replication transcriptional complexes, resulting in the increased expression of the autophagy marker LC3-II and the elevation of the double-membrane vesicle (DMV) structure [121]. Previous studies have reported that the endoplasmic reticulum proteins VMP1 and TMEM41B, which are important for viral replication, play crucial roles in DMV formation. These proteins are involved in autophagy and lipid metabolism [122]. Therefore, SADS-CoV infection induces autophagy and utilizes these proteins to promote DMV formation, ultimately facilitating viral proliferation.

## 6. Swine Acute Diarrhea Syndrome Coronavirus (SADS-CoV) and Hosts’ Critical Factors

Coronaviruses infect host cells by exploiting the host cell’s functional units and metabolism to promote their own proliferation [123]. We refer to the functional units of the host cell capable of exerting a significant influence on coronavirus infection and proliferation as host critical factors. Examples of such functional units include the angiotensin-converting enzyme (ACE2) and dipeptidyl peptidase-4 (DPP4), which are found in the cell membrane [124,125]. These host factors are known to be critical for virus-infected cells, and their study is essential for the identification of potential drug targets in viral design. Recent research has focused on identifying the key host factors that vary across different coronaviruses that infect the cells of various species [126,127,128]. The screening process for SADS-CoV host critical factors has revealed the presence of these factors throughout the viral proliferation process [129].

Studies on PDCoV have identified solute carrier family 35 member A1 (SLC35A1) as a key molecule in the salivary acid (SA) synthesis pathway [130]. Knockdown of the *SLC35A1* gene leads to a reduction in the cell surface salivary acid (SA), subsequently causing a decrease in viral adsorption on target cells [131]. Both SADS-CoV and PDCoV exhibit a similar dependence on SA, as they primarily affect the early stages of cellular infection by these viruses [132]. The consistent deletion of the *SLC35A1* gene reduces the infectivity of SADS-CoV, suggesting that *SLC35A1* may be a key host factor for SADS-CoV infection [130].

A significant portion of the key host factors is focused on the interaction process with the SADS-CoV spike protein [53]. Viral invasion and proliferation involve a modification of the S protein and the cleavage of specific sites, which facilitate membrane fusion and other related processes [133]. Furin protease is responsible for hydrolyzing the S1/S2 cleavage site and the 97 aa cleavage site upstream of it in the SADS-CoV S protein, and this process is necessary for viral S protein-mediated cell–cell fusion [133]. In addition, peptidylprolyl isomerase B (PPIB), a member of the proline isomerase family, binds to the receptor-binding domain of the SADS-CoV S protein [134]. Vimentin interacts with the SADS-CoV S1 subunit, contributing to essential cellular processes such as cell division and migration [135]. A reduction in PPIB and vimentin expression induced by RNA interference significantly inhibited the replication of SADS-CoV [134]. However, the detailed mechanisms of action of these two key host factors remain unknown [134]. Bile acids (BAs) play an important role in viral invasion [136]. Cholic acid (CA) in a single BA promotes SADS-CoV infectivity during the early stages of SADS-CoV infection by modulating lipid rafts and the membrane cholesterol-mediated endocytosis of dynamin 2, which is associated with caveolae-like invaginations of cell membranes, facilitating viral entry into cells [136]. Viral activity in the host cells is crucial for the proliferation of daughter viral particles. During this stage, various host factors interact with the virus, influencing its behavior.

The modes of action of the key host factors at this stage can be classified into two categories. The first category primarily affects the replication and transcription of the viral genome/subgenome. PLAC8, a cysteine-rich protein widely distributed in eukaryotes and highly expressed in the placenta, lung, and intestine, is involved in autophagy and epithelial-mesenchymal transition (EMT) pathways [137,138], which are essential for CoV infection [139]. The deletion of the PLAC8 protein during SADS-CoV proliferation in host cells affects viral translocation and the formation of DMVs [115,140], resulting in a decrease in viral subgenomic RNA expression and hysteresis, effectively blocking SADS-CoV infection [139]. Additionally, ZD17, a cytoplasmic enzyme with palmitoylation activity, is crucial for SADS-CoV’s genomic RNA replication [141]. The second class of critical host factors promotes autophagy to facilitate viral replication. The SADS-CoV infection of Vero E6 cells induces complete autophagy, with the upregulation of ATG5, an essential protein for autophagosome formation, and LC3-II [115,117,142], an autophagy marker [93]. The inhibition of ATG5 prevents SADS-CoV replication, suggesting that SADS-CoV-induced autophagy is dependent on ATG5. SADS-CoV infection indirectly regulates *ITGA3* expression and inhibits the Akt/mTOR pathway to induce autophagy [118] (Figure 3).

None of the previously mentioned host critical factors were involved in promoting viral replication through the innate immunosuppression pathway in the current study. Only TET2, a protein expressed by host cells, has been reported to regulate SADS-CoV entry into cells by inhibiting the host cell immune pathway [143]. The mechanism of action of certain key host factors associated with SADS-CoV infection remains unclear. For example, RHOA [144,145], PRL18 [146,147,148], and RALY [149], which were identified as host factors interacting with SADS-CoV M, affected viral proliferation upon overexpression. These three proteins may be involved in viral RNA processing, protein processing, and modification, based on additional related research [150].

## 7. Swine Acute Diarrhea Syndrome Coronavirus (SADS-CoV) Prevention and Treatment Tools

Efficient virus detection is pivotal in disease prevention within practical livestock production activities. Various methods for detecting SADS-CoV have been elucidated in the literature, encompassing polymerase chain reaction (PCR) diagnostic tests at the genomic level and serologic tests at the protein level. PCR detection technology, particularly a single real-time reverse transcription-polymerase chain reaction (RT PCR) assay grounded in TaqMan [151] and SYBR green probes [152], has gained widespread acceptance. Multiplexed RT-PCR assays have also been successfully established and tailored for SADS-CoV, TGEV, PEDV, and PDCoV [153]. Innovative strategies have emerged to address concerns of cross-reactivity in multiplex PCR assays. The incorporation of artificially designed internal positive control nucleic acid fragments, absent in the analyzed pathogens or host species, has significantly bolstered confidence in these assays [154]. Furthermore, the evolution of isothermal nucleic acid amplification methods offers a more accessible avenue for SADS-CoV detection in practical production [155]. This includes detection methods such as loop-mediated isothermal amplification method (LAMP) technology and a microfluidic reverse transcription loop-mediated isothermal amplification method (RT-LAMP) microarray system [156], combining microfluidic microarray technology and thermostatic PCR technology alongside single-tube multiplexed LAMP technology coupled with the CRISPR/Cas12a system [157], allowing field deployment and visual detection.

Current assays are evolving in pursuit of enhanced detection efficiency, specificity, and visualization. Although enzyme-linked immunosorbent assay (ELISA) assays for SADS-CoV, utilizing recombinant S proteins and serum samples [158], have been reported, their application remains confined to laboratory settings.

The availability of the SADS-CoV/CN/GDWT/2017-P83 attenuated strain is crucial for developing an effective vaccine against SADS-CoV. This strain was obtained through the serial propagation of the SADS-CoV/CN/GDWT/2017 strain in Vero cells for 83 generations. This strain exhibits 10 non-silencing mutations in various genes, ORF1a/1b, S, E, NS3a, M, and N, compared with the original strain. Additionally, there is a 58 nt deletion in NS7a/b [12,27], resulting in a modification of 38 amino acids in the NS7a protein and a loss of NS7b expression [34]. The attenuating effect of treatment was observed at both the infected tissue and individual levels. Specifically, at the tissue level, the SADS-CoV/CN/GDWT/2017-P83 strain was detected only at low copy numbers in the ileum and mesenteric lymph nodes, reflecting reduced tissue eosinophilia. Additionally, the strain did not cause clinical signs of any thinning of the intestinal wall, impaired permeability, or an accumulation of significant quantities of yellow and watery feces in the ileum and mesenteric lymph nodes. At the individual level, piglets inoculated with SADS-CoV/GDWT-P83 demonstrated mild and temporary diarrhea without the typical onset of watery diarrhea [85].

RNA interference (RNAi) has emerged as an effective antiviral therapy capable of impeding viral replication in vitro and in vivo. The pMulti-shRNA-M-mCherry plasmid system, derived from pSpCas9(BB)-2A-mCherry, targets the highly conserved M gene of SADS-CoV and demonstrates robust antiviral activity at the cellular level by expressing multiple short hairpin RNAs (shRNAs). A 99.4% reduction in SADS-CoV genomic RNA was observed in multi-shRNA-expressing cells, compared to that in non-transfected control cells [159]. The SADS-CoV M protein yield significantly decreased in multi-shRNA-expressing cells. Viral infection did not cause any cytopathic effect (CPE) in cells, and their viability returned to normal levels. This therapeutic approach uses an RNAi system with multiple shRNAs to address the limitations of single shRNA expression vectors in preventing viral infections. First, multiple shRNAs can reduce the limitations of poor therapeutic efficacy, caused by viruses evading the RNAi recognition mechanism by accumulating mutation sites [160]. Second, the designed target is located on the M gene, which is highly conserved in coronaviruses and is indispensable for the viral life cycle, ensuring the effectiveness and stability of the target. However, the selection of an appropriate carrier for delivery to the body is an important consideration in practical production applications [159].

Drug screening has resulted in the strategy of drug repurposing for the treatment of SADS-CoV infection. Gemcitabine [161], mertiomacrophenol ester [162], mycophenolic acid [163], methylene blue [164], and chikungunya have all demonstrated inhibitory effects on SADS-CoV in a dose-dependent manner with low toxicity. The mechanism of viral infection inhibition by these five drugs has been determined. Both chikungunya and methylene blue inhibit viral entry into the host. Chikungunya blocks cell–virus binding by targeting the host cells. Methylene blue exerts dual effects on cells and viruses [165]. It can directly inactivate viruses and inhibit SADS-CoV infection by disrupting virus–cell interactions before viral entry. Gemcitabine [166], mertiomacrophenolate (MPA), and mycophenolic acid increase cellular activity by inhibiting viral replication post cellular entry. MPA induces the expression of interferon-stimulated genes (ISGs) and acts synergistically with interferons to inhibit hepatitis C virus (HCV) replication. However, whether MPA inhibits the intracellular replication of SADS-CoV by using a similar mechanism to inhibit the intracellular replication of the HCV remains to be explored [161].

Natural medicine screening is an avenue of research being pursued for the treatment of SADS-CoV [167]. Emodin has broad-spectrum antiviral activity against SADS-CoV throughout its replication cycle, without directly affecting the infectivity of the virus [168]. Its antiviral effect is primarily realized by reducing the attachment of viral particles to the cell surface. This antiviral effect is most pronounced during the attachment stage. Furthermore, emodin has been shown to activate the TLR3-IFN-λ3-ISG15 pathway in certain cells [169], which reduces SADS-CoV infection by modulating the immune response in host cells [170]. Gossypol (GOS) [171,172], another natural product, has demonstrated inhibitory effects on SADS-CoV infection in vitro and in vivo. GOS inhibits the RNA-dependent RNA polymerase (RdRp), a crucial enzyme involved in viral replication. This mechanism is similar to that of the small-molecule drugs, raltegravir and monoravir [173], which are currently used in neo-coronary therapy. However, unlike raltegravir and monoravir, GOS has an altered site that affects the binding of RdRp to non-substrates. This mode of action effectively overcomes the limitations of nucleoside analogs in the prevention and control of coronaviruses. For example, the replication–proofreading function of ExoN is resistant to RdRp inhibitors [174,175].

## 8. Discussion

SADS-CoV, an α-coronavirus with structural similarities to β-coronaviruses, has been extensively studied and found to exhibit several unique characteristics. One of these features is the ability to reduce immunogenicity by aligning the spike protein trimer conformation more tightly and making flexible structural adjustments [46]. Another characteristic of SADS-CoV is its reliance on complete autophagic [94,116,117] flux to increase viral copies. This suggests that there may be genome crossover between different species of the genus coronavirus [33,34], leading to evolutionary changes and the emergence of novel coronaviruses with distinct characteristics.

The temporal gaps and spatial uncertainty observed in epidemiological studies on the virus suggest a high degree of latency. Therefore, a high level of vigilance and continuous monitoring of swine farms, especially those previously affected by other swine coronaviruses, is imperative to minimize the economic impact of outbreaks and enhance our understanding of the evolutionary and epidemiological attributes of the virus. In addition, extensive cytophilicity and interspecies transmission of the virus [25] may have accelerated its evolution. Human-infecting coronaviruses typically have natural hosts and exist intermediate hosts before transmission to humans. Their evolutionary progression can be categorized into three stages: animal-only, zoonotic, and human-specific viruses. Recent outbreaks of SARS-CoV in 2003 [65,176,177] and MERS-CoV in 2012, which originated in bats, have demonstrated that the virus can only infect humans through the evolution and adaptation of intermediate hosts, such as civets and camels [178,179]. Predicting the possible mutation sites of SADS-CoV is crucial for understanding its potential evolution and assessing the risk of its transmission to humans.

Understanding viruses and the mechanisms of virus–host interactions, particularly with regard to SADS-CoV, is of great scientific and practical importance. While previous research has aided our understanding of the key factors of the virus’s hosts using integrated bioinformatics analysis, key host proteins that interact directly with the virus to completely block SADS-CoV invasion have not been identified [12,53]. This may be due to the use of immortalized cell lines in existing studies, which may have partial gene deletions or diverse mechanisms of cell invasion caused by the virus. Currently, established library screening methods typically use a limited number of infection pluralities to avoid false positive experimental results caused by the multiple knockdown of individual cells [180]. The potential key factors screened were all single genes. The functions and mechanisms of these single genes have been verified [130,139,141]; however, no studies have been conducted on the combined effects of multiple genes. Therefore, further research is needed to validate the results of library screening for co-interactions of multiple genes.

Vaccine research is a key initiative aimed at preventing viral infection. Despite ongoing efforts to screen for strains with attenuated activity, the traditional vaccine development process remains arduous and carries the risk of infection. mRNA vaccines have proven to be highly advantageous in addressing outbreaks caused by novel coronaviruses in the human population [181,182]. This vaccine’s characteristics can be summarized as “short development cycle, no risk of infection, simple production process, dual immunity mechanism, high immunogenicity, and no need for adjuvants” [183]. Vaccine development for SADS-CoV may have significant implications for future mRNA vaccine development during sudden outbreaks.

## 9. Conclusions

Coronaviruses persist as a formidable threat to human society, showcasing their capacity to traverse species boundaries and profoundly impact human health. While the prospect of direct human infection with porcine coronaviruses remains low, these pathogens exert an indirect influence by disrupting the agricultural sector, particularly the farming industry. Notably, the emergence of swine acute diarrhea syndrome coronavirus (SADS-CoV) in recent years has triggered outbreaks in various provinces in southern China. Despite occurring in manageable outbreaks, the virus merits heightened attention due to its novel structural attributes and enhanced cellular invasion capabilities, as evidenced by an expanding body of research.

While prior studies have delved into the genomic, structural, evolutionary, and epidemiological facets of SADS-CoV, the virus’s elusive receptor remains an enigma. The absence of a known receptor, coupled with a commercially available vaccine, underscores the imperative for ongoing research efforts to unravel the intricacies of this viral threat. This review systematically consolidates the existing body of research on SADS-CoV since its initial report, offering comprehensive insights as a valuable reference for future investigations. As we confront the challenges posed by this emerging coronavirus, continued exploration and a deeper understanding are essential to pave the way for effective preventive and therapeutic interventions.

## Figures and Tables

**Table 1:** SADS-CoV susceptible cell lines [25,26,92].

Host	Cell Line	Origin of Cell Line
Human	Huh-7	*H. sapiens* liver gallbladder
	HepG2/C3A	*H. sapiens* liver
	293T	*H. sapiens* embryonic kidney
	A549	*H. sapiens* lung
	HeLa	*H. sapiens* cervix
	RD	*H. sapiens* embryonic
	Hep2	*H. sapiens* larynx
Bat	BFK	*Myotis petax* kidney
	Tb-1	*Tadarida brasiliensis* lung
	RaLu	*Rhinolophus affinis* lung
	RsHe	*Rhinolophus sinicus* heart
	RlKi	*Rousettus leschenaultia* kidney
	PaKi	*Pteropus alecto* kidney
Mouse	NIH/3T3	*Mus musculus* embryo
	RAW 264.7	*Mus musculus* monocyte/macrophage
	L929	*Mus musculus* lung
Hamster	BHK-21	*Mesocricetus auratus* kidney
	CHO	*Chinese hamster* ovary
Rat	BRL	*Rattus norvegicus* liver
	NRK-52E	*Rattus norvegicus* kidney
Swine	ST	*S. scrofa* testicle
	PK15	*S. scrofa* kidney
	LLC-PK1	*S. scrofa* kidney
	IPEC-J2	*S. scrofa* intestine
	SIEC	*S. scrofa* intestine
	IBRS	*S. scrofa* kidney
Monkey	Marc-145	*Cercopithecus aethiops* kidney
	Cos-7	*Cercopithecus aethiops* kidney
	BSC-40	*Cercopithecus aethiops* kidney
	Vero	*Chlorocebus aethiops* kidney
	LLC-MK2	*Macaca mulatta* kidney
Cat	FK	*Felinae* kidney
Dog	MDCK	*Canis familiaris* kidney
Mink	Mv.1.Lu	*Mustla vison* lung
Chicken	DF-1	*Gallus gallus* embryo
Gerbil	Primary kidney cells	

Note: Indicators of SADS-CoV-susceptible cells include efficient viral replication, antigen expression, and cytopathic effect (CPE).

## Data Availability

No new data were created or analyzed in this study. Data sharing is not applicable to this article.
